# Non-Darwinian Molecular Biology

**DOI:** 10.3389/fgene.2022.831068

**Published:** 2022-02-16

**Authors:** Alexander F. Palazzo, Nevraj S. Kejiou

**Affiliations:** Department of Biochemistry, University of Toronto, Toronto, ON, Canada

**Keywords:** neutral theory, evolution, junk DNA, junk RNA, robustness

## Abstract

With the discovery of the double helical structure of DNA, a shift occurred in how biologists investigated questions surrounding cellular processes, such as protein synthesis. Instead of viewing biological activity through the lens of chemical reactions, this new field used biological information to gain a new profound view of how biological systems work. Molecular biologists asked new types of questions that would have been inconceivable to the older generation of researchers, such as how cellular machineries convert inherited biological information into functional molecules like proteins. This new focus on biological information also gave molecular biologists a way to link their findings to concepts developed by genetics and the modern synthesis. However, by the late 1960s this all changed. Elevated rates of mutation, unsustainable genetic loads, and high levels of variation in populations, challenged Darwinian evolution, a central tenant of the modern synthesis, where adaptation was the main driver of evolutionary change. Building on these findings, Motoo Kimura advanced the neutral theory of molecular evolution, which advocates that selection in multicellular eukaryotes is weak and that most genomic changes are neutral and due to random drift. This was further elaborated by Jack King and Thomas Jukes, in their paper “Non-Darwinian Evolution”, where they pointed out that the observed changes seen in proteins and the types of polymorphisms observed in populations only become understandable when we take into account biochemistry and Kimura’s new theory. Fifty years later, most molecular biologists remain unaware of these fundamental advances. Their adaptionist viewpoint fails to explain data collected from new powerful technologies which can detect exceedingly rare biochemical events. For example, high throughput sequencing routinely detects RNA transcripts being produced from almost the entire genome yet are present less than one copy per thousand cells and appear to lack any function. Molecular biologists must now reincorporate ideas from classical biochemistry and absorb modern concepts from molecular evolution, to craft a new lens through which they can evaluate the functionality of transcriptional units, and make sense of our messy, intricate, and complicated genome.

## Introduction

We live in the post genomic era. Our ability to analyze nucleic acid sequences has increased many orders of magnitude in the past 2 decades, largely due to the advent of high throughput sequencing technology. This has allowed us to undertake large-scale molecular biology experiments unimaginable a few decades ago. For example, we are now able to isolate and sequence long non-coding RNAs that are present at a few copies per thousand cells. Our newfound ability to analyze DNA and RNA sequences has been exploited by large consortia, such as the ENCODE project, that have allocated significant resources to catalogue every biochemical event associated with the entire genome (ENCODE Project Consortium et al., 2007; ENCODE Project Consortium et al., 2012). Despite these developments, the molecular biology community seems adrift. This is exemplified by many individuals within the community who discover that a segment of non-coding DNA has some function, then falsely extrapolate their finding to the genome at large and erroneously conclude that junk DNA is in fact a vast repository of dark matter (“matter”, here being understood as a collection of poorly defined functional entities that have yet to be elucidated). In terms of advancing large scale theories about genome organization, or the contribution of lncRNAs to cellular homeostasis and organismal development, new insights are few and far between. To paraphrase Sydney Brenner, many in the field are drowning in a sea of data and starving for knowledge (Brenner, 2003).

## How Did We Get to This Point?

From the 1850s to the 1950s, biochemistry had made spectacular advances in understanding cellular life. Despite this, the biochemical and molecular processes that explained heredity remained mysterious. It wasn’t until 1953, with the elucidation of the structure of DNA, that a coherent molecular basis for heredity could be formulated. DNA contained sequence information where the series of nucleic acid bases along one polymer chain specifies the sequence of nucleic acid bases along a second polymer chain (Watson and Crick, 1953). Biological information was digital, each quantum being a nucleotide (C, A, T or G). This sequence in nucleic acids could then specify the order of other polymers, such as proteins (Crick, 1958).

Before the double helix, the biochemistry of life was investigated as a series of reactions. Afterwards, life was seen as being imbued with molecules that could carry information. With this shift, the budding field of molecular biology described these new molecules and their properties using terminology (e.g., sequence, transcription, translation, codon, frame) that could have been lifted from computer science and information theory, two disciplines that were also developing at the same time.

The degree to which the emphasis had shifted from investigating cellular activity through the lens of enzymology to that of information is nicely illustrated by a quote from Sydney Brenner (about the problem of co-linearity between DNA and protein sequence) (Wolpert, 1994):

At that time, the biochemists of the world were preoccupied by where you get the energy to make the proteins, and we had to spend weeks, months, saying “don’t worry about energy, energy will look out for itself. The important thing is how to get everything in the correct order. How do you get everything specified in this order, that is, the genetic code.” I think that this is such an important and fundamental divergence from anything else in Biology, that it is a total discontinuity, at least this is the way I’ve seen it. And it has of course constrained quite a lot of later developments in biology. And of course it crystalizes the types of problems you have to solve in a clear-cut way, because now they do not remain, sort of, vague problems that you can ask sort of rhetorical problems about. But you can actually sit down and ask, “If I had a gene and I could do the fine structure, and if I had a protein that I could sequence, then I could show whether the gene was co-linear.”

In the following decades molecular biologists fleshed out the mechanistic details of how information was duplicated and how it specified the synthesis of functional molecules such as proteins.

## The Molecular Evolution Revolution

The molecular basis of heredity not only changed our view of cellular and organismal activity, but also altered our understanding of evolution. In the first half of the 20th century, the theoretical basis of how mutations spread within a population were developed by population geneticists such as J.B.S Haldane, Sewall Wright and Ronald Fisher. By the 1950s, it was widely accepted that evolution occurred through natural selection at the genetic level. Given that the molecular basis for inheritance could now be linked to the sequence of bases in DNA, and this could be further extrapolated to the sequence of amino acids in proteins, it was unsurprising that some molecular biologists started to tackle evolutionary questions in the mid-1960s.

In 1962, Emile Zuckerkandl, under the supervision of Linus Pauling, found that the number of amino acid differences between the hemoglobin proteins isolated from any two species, correlated with how long ago they had diverged from a common ancestor (as per paleontological estimates)—a concept later dubbed the “molecular clock” (Zuckerkandl and Pauling, 1962; Margoliash, 1963; Zuckerkandl and Pauling, 1965). This established that species relatedness could be determined at the molecular level, and that protein paralogues evolve at a constant rate in different lineages. Around the same time, Jack Hubby and Richard Lewontin, used gel electrophoresis to demonstrate that different individuals within a species had a surprisingly high amount of variability in any given protein (Hubby and Lewontin, 1966; Lewontin and Hubby, 1966). The variability was far greater than what most population genetics models had predicted. Others had noted that many of these substitutions led to mostly neutral changes in proteins (Freese, 1962; Margoliash, 1963). All of these observations led to a crisis in evolutionary biology.

This crisis was further deepened by Motoo Kimura’s 1968 publication “Evolutionary Rate at the Molecular Level” (Kimura, 1968). The amount of protein variation between and within species, estimated by the new molecular biology techniques, inferred such a high substitution rate that if they all consisted of alleles that were under selection, the cost of replacing these alleles (in “genetic deaths”) would be intolerable. Instead, Kimura proposed that the majority of these mutations must be neutral. Furthermore, Kimura demonstrated that slightly deleterious or beneficial mutations behave like neutral mutations, provided that the absolute value of their selection coefficient was smaller than the inverse of the effective population size.

$$\left|s\right|<1/N_{e}$$

In this equation, the selection coefficient (*s*) is the average decrease or surplus in offspring in the mutant compared to a wildtype individual, and the effective population size (*N*_*e*_) is the number of breading individuals that randomly mate in a species. In practice, *N*_*e*_ is much lower than the actual population size as it is dependent on a number of different parameters (Lynch, 2007). In humans, the effective population size has been estimated to be about 10,000 which is a bit lower than the typical number for most other animals (Charlesworth, 2009). If a mutation falls within the parameters of the above equation, it does not clear what Michael Lynch called “the drift barrier” (Lynch et al., 2011; Sung et al., 2012), and its fixation will be dictated by neutral evolution.

A year after Kimura’s paper, evolutionary biologist Jack King and Thomas Jukes independently established a similar conclusion (King and Jukes, 1969); however, many of their arguments relied on the biochemistry of proteins and the types of mutations seen in nature. Amino acid substitutions between species and allele variants within species occurred in regions that were less important (for example away from the active site of enzymes). These changes were typically conservative (swapping amino acids of similar biochemical nature), and rarely altered the activity of a protein. Moreover, the substitution rate was lower in genes that play fundamental roles in cell physiology. In the next few decades, important advances in the neutral theory were further developed by Kimura and his colleague Tomoko Ohta (Kimura and Ohta, 1974; Ohta, 2003). All of this data and analysis argued that most evolution was due to random neutral genetic drift at the molecular level.

At the time, natural selection was believed to be the main driver of evolution and the new neutral theory caused considerable controversy within the field–creating what is commonly referred to as the neutralist-selectionist debate (Nei, 2005). Eventually, DNA sequencing data validated predictions made by neutral theory, namely that the rate of fixation is higher in parts of the genome with low functional constraint; such as pseudogenes and introns (Kimura, 1991). It became increasingly difficult to justify why natural selection would maintain high rates of mutations in these genomic regions. Today, when researchers claim that a portion of a protein is important because it is conserved, they are in fact using arguments (whether they know it or not) that originated in the neutral theory of molecular evolution (Hughes, 2007).

Of course, mutations are not simply nucleotide or amino acid substitutions. INDELs, small and large, would be subject to the forces of neutral and near-neutral evolution. Geneticist Susumu Ohno, who investigated gene duplication and its evolutionary consequences, proposed that newly duplicated genes either gained new functions (neofunctionalization) or lost function entirely, becoming pseudogenes (Ohno, 1972a). Ohno also recognized that the genome contained other non-functional entities, such as short repeats and that as much as 90% of the genome was likely non-functional (Ohno, 1972b). He called this “junk DNA”, a term that had been previously used colloquially in molecular biology circles (Graur, 2013).

To add fuel to the fire, in the 1970s the contribution of neutral evolution to phenotypic change was explored (Lande, 1976; Nei, 2013), although some of these ideas had been previously advanced by Sewall Wright (Wright, 1932). The idea that natural selection may not be the only mechanism for phenotypic evolution was further popularized by Lewontin and Stephen J. Gould. They warned those who studied phenotypic evolution that many of their explanations were “just-so stories” and that other factors may explain how any given phenotypic trait evolves (Gould and Lewontin, 1979). This includes not only neutral evolution, but also developmental constraints, pleiotropy and historical contingencies. Even today, the degree to which neutral evolution impacts phenotypic changes is poorly understood (Zhang, 2018).

The neutralist-selectionist debate is still ongoing. Some population geneticists in the selectionist camp have advocated that most mutations can reach fixation because they are linked to nearby alleles that are under positive selection, so called evolution by draft (Gillespie, 2000; Kern and Hahn, 2018). However, these arguments do not invalidate the idea that most mutations *by themselves* are nearly neutral, the main thesis of the neutralist camp. Indeed, as pointed out by several commentators, rampant draft, if anything, lowers the effective population size and would thus further erode natural selection’s ability to weed out slightly deleterious mutations (Jensen et al., 2019). Other factors, such as fluctuations in selection coefficients between generations can further diminish the power of natural selection to differentiate between slightly deleterious or beneficial from neutral mutations (Nei, 2013). Although many details about molecular evolution are still debated, the main tenants of the neutral theory have been confirmed by numerous studies. Predictions made by the neutral theory match closely what is seen in countless analyses of single nucleotide polymorphism (SNP) distributions and comparative genomic surveys (Lynch, 2007; Koonin, 2011; Nei, 2013; Graur et al., 2016).

## Molecular Biology: Drowning in a Sea of Data and Starving for Knowledge

Although neutral theory has made important intellectual advances, and was fueled by advances in molecular biology, many life scientists are either unaware of these developments or have failed to assimilate these new concepts. As the spiritual descendants of Watson and Crick, most modern molecular biologists assume that DNA *is* information. This is implicit when they use the shorthand of “sequence” for a short stretch of DNA. And for the decades following the elucidation of the double-helical structure, there was little reason to deny this view given the furious pace at which researchers were discovering molecular machines that would copy, read, and translate the code-script. Despite this, there were signs that the human genome may contain vast tracts of non-functional DNA that could harbor neutral mutations. First, several researchers, using concepts from population genetics, estimated that the human genome contained about 30,000 genes with an upper limit of 40,000 (Muller, 1966; King and Jukes, 1969; Ohno, 1972a; Hatje et al., 2019). Since the average protein size was known (about 50 kDa) this meant that most of the genome had to be non-coding. At the same time, it was observed that eukaryotic genomes contained a large fraction of repeated sequence (Britten and Kohne, 1968), and that much of this was composed of transposable elements that were unlikely serving any role for the organism but instead were the product of selfish DNA (Doolittle and Sapienza, 1980; Orgel and Crick, 1980). Then with the discovery of splicing, it was realized that much of the genome was transcribed into introns that were discarded soon after they were synthesized (Berget et al., 1977; Chow et al., 1977; Gilbert, 1978; Berk, 2016). Finally, during the completion of the human genome project, it was revealed that nearly 98% of the genome was non-coding. While many researchers were surprised (Hatje et al., 2019), this observation was entirely consistent with earlier findings and gene estimates.

One of the problems with accepting that the human genome consists mostly of junk DNA, is that additional functional DNA elements, beyond protein-coding genes, were widely known since the 1960s (although many researchers continue to state, erroneously, that *all* non-coding DNA was once thought to be junk). Leaving aside the fact that nearly a quarter of the genome consists of intronic sequence, it has been long known that genomes contain promoters (Ippen et al., 1968), enhancers (Banerji et al., 1983; Gillies et al., 1983; Mercola et al., 1983), silencers (Abraham et al., 1983), origins of replication (Beach et al., 1980), telomeres (Blackburn and Gall, 1978), centromeres (Clarke and Carbon, 1980), and a wide array of genes transcribed into various non-coding RNAs, including rRNA (Roberts, 1958; Morrow et al., 1974), tRNAs (Holley et al., 1965), snRNAs (Yang et al., 1981), and other non-coding RNAs (Stark et al., 1978; Walter and Blobel, 1982; Brannan et al., 1990; Brockdorff et al., 1992). Although many of the founders of molecular biology were quite open to the idea of junk DNA, many of the molecular biologists that came afterwards had hyper-adaptationist tendencies. With the discovery of every new functional non-coding RNA, these researchers would extrapolate, erroneously, this finding to all cryptic transcription and conclude that it is all functional. With the discovery that a given transposable element was co-opted for a functional purpose, these molecular biologists would conclude that all transposable elements have some hidden function. The rejection of junk DNA, in the mind of these researchers, led to the idea that the genome consisted of mostly “dark matter”.

The pinnacle of this line of reasoning came with the Encyclopedia of DNA Elements, or ENCODE project, whose goal was to map all of the biochemical activities in the genome (ENCODE Project Consortium et al., 2007; ENCODE Project Consortium et al., 2012). Although the initial ENCODE publications, which documented all the biochemically active sites in 1% of the human genome, interpreted the data very conservatively, the second round of publications, which examined the entire genome, famously did not. The most publicized “conclusion” from this work was that at least 80% of the genome had some function (ENCODE Project Consortium et al., 2012), again implying that junk DNA did not exist (Pennisi, 2012). Embedded in the collection of ENCODE papers was a compilation of all the messy reactions that we now have come to accept as commonplace in eukaryotic genomes. Focusing on the RNA results, it was seen that most of the genome was transcribed, albeit at some low level, that transcripts often began and ended at a wide variety of locations with many of these originating from intergenic regions, and that much of the transcriptional patterns were cell-type specific (Djebali et al., 2012). Similar findings were seen for other biochemical events that could be spatially mapped back to the genome (Spitz and Furlong, 2012; Thurman et al., 2012). This includes promoter-like regions, which appeared to outnumber protein-coding genes by an order of magnitude, and transcription factor binding sites, which were present throughout the genome, irrespective of whether they were near any genes. In response to these unsupported conclusions, other researchers pointed out that the new ENCODE results were entirely consistent with a junky, messy genome that is predicted by our modern understanding of molecular evolution (Eddy, 2012; Doolittle, 2013; Eddy, 2013; Graur et al., 2013; Niu and Jiang, 2013; Doolittle et al., 2014; Palazzo and Gregory, 2014; Ponting, 2017).

The problem is that when many molecular biologists are confronted with the idea that much of the genome, especially junk DNA, may be fixed by drift, they counter that natural selection would not tolerate waste or useless junk. They then use a few counterexamples, for example the presence of a functional non-coding RNA, as evidence against junk DNA. These conclusions are, of course, an over-extrapolation. Part of the problem is that by equating DNA to information, molecular biologists implicitly assume that all DNA is functional. Making the situation worse, many molecular biologists incorrectly equate natural selection with evolution. Biological function, however, implies that the trait, or element in question is currently under selective constraint (Doolittle et al., 2014; Linquist et al., 2020), and as described above, less than 10% of the human genome is under selection (Lindblad-Toh et al., 2011; Ward and Kellis, 2012; Palazzo and Gregory, 2014; Rands et al., 2014; Graur, 2017).

To move forward, the molecular biology community must not only gain a deeper appreciation of how evolution works on the molecular level, but also reincorporate concepts from biochemistry. Indeed, King and Jukes, two of the instigators of the molecular evolution revolution recognized this (King and Jukes, 1969). Their critique of pan-adaptionalist thinking was partially made on biochemical grounds. They noted how observed changes in proteins, either between paralogs in different organisms or due to polymorphisms within an organism, were mostly due to conservative substitutions between chemically similar amino acids in parts of the protein which could tolerate such changes. For these reasons they argued that much of evolution was non-Darwinian.

**FIGURE 1:**
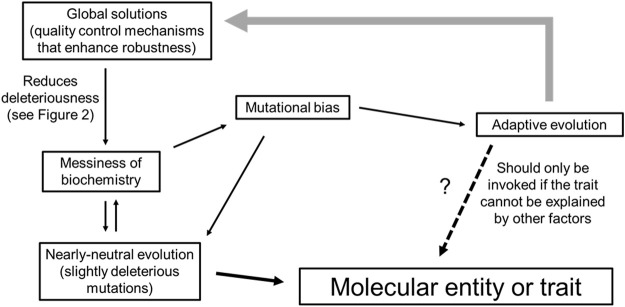
The mechanisms that shape traits in a weak selection regime.

In the next few sections, we will cover some of the modern ideas and concepts from both biochemistry and molecular evolution, and try to build a more modern conceptual framework for molecular biology that is non-Darwinian in outlook. First, we discuss how cellular biochemistry is imperfect, by virtue of the chemical properties of macromolecules, and how genetic drift hinders their improvement and further exacerbates their messiness, especially in eukaryotes. Next, we consider how eukaryotic systems evolve robust quality control systems (“global solutions”) which have a two-fold effect of ameliorating numerous entities that experience slightly deleterious mutations (“local problems”), while also permitting the proliferation of additional cellular sloppiness. Then, we discuss mutational bias which drastically affects the path of genome evolution. Only by incorporating concepts of biochemical sloppiness, global solutions and mutational bias into neutral evolution, we can propose a null hypothesis for adaptive evolutionary claims (Figure 1). Lastly, we discuss how neutral evolutionary processes can increase the complexity of an organism which may in turn permit the evolution of novel adaptive traits.

## Cells are Sloppy

The bench scientist has an intuitive notion that biochemistry is imperfect. Many restriction enzymes that supposedly cut only one sequence also have weak activity on others (Wei et al., 2008). RNA binding domains that supposedly recognize one unique RNA motif, will bind almost any other RNA molecule when tested in isolation (Jankowsky and Harris, 2015), with apparent affinity for one motif only readily observed when they are presented with a mixture of RNAs (Ray et al., 2009). Of course, these results are due to the varying affinities a given RNA binding domain has for different RNA sequences. And these imperfections extend far beyond the test tube and the cellular environment. Clinicians and drug developers actively rely on the sloppiness of enzymes to interact with compounds never encountered in the natural world. Small molecules that are never seen in nature are nevertheless acted on by a cadre of cellular enzymes, such as in cytochrome p450 family members (Atkins, 2020). Even in the absence of drugs, enzymes still engage in a variety of adventitious interactions with non-canonical protein partners or endogenous metabolites (Khersonsky and Tawfik, 2010; Tawfik, 2020). This is in part a consequence of the nearly-neutral theory of molecular evolution, that the underlying mutation which allows enzyme promiscuity is not sufficiently deleterious to be selected against. As a result, enzymes accumulate slightly deleterious mutations and as a result protein activities and interactomes become even more messy and error-prone (Levy et al., 2009). This is also due to basic principles of chemistry, that non-canonical reactions will occur, and the degree that these happen, as opposed to the most favorable reaction, is dictated by the Boltzmann factor between the two competing states:

$$\frac{P_{1}}{P_{2}}=e^{\left(E_{1}-E_{2}\right)/kT}$$

In this equation, the ratio of the probabilities of two competing states (*P*_*1*_ and *P*_*2*_) are dictated by the energies associated with each state (*E*_*1*_ and *E*_*2*_), the Boltzmann constant (k) and the temperature (*T*). The greater the energy differential is, the more skewed the ratio will be. However, the probability of the unfavored state can never be zero as this would require an infinite energy differential. Although the Boltzmann factor is used to describe the population of two states at equilibrium, it can also be used to describe competition between two reactions, with *E*_*1*_ and *E*_*2*_ representing the activation energy of the competing reactions, although there are some differences (Sawato et al., 2019). Using Boltzmann distributions, one can use energetic differentials between tautomeric states of nucleic acid bases and their propensity to base pair, to predict some aspects of nucleotide misincorporation during DNA replication (Kimsey et al., 2015; Kimsey et al., 2018).

Focusing in on gene expression, messiness can be seen at every step. Nucleotide misincorporation during transcription and amino acid misincorporation during translation, which can be as high as 10^−3^ per codon (Kramer and Farabaugh, 2007), will produce proteins that differ from their canonical sequence that further contribute to biochemical sloppiness (Drummond and Wilke, 2009). Indeed, with error rates this high, a protein such as RanBP2/Nup358, which contains 3,224 amino acids, will have on average one amino acid misincorporation error for every molecule in a human cell. This messiness is not only true for simple molecular interactions and reactions, but for more complicated processes. Many random pieces of DNA can activate transcription (Reinke et al., 2008; Gerber et al., 2013; White et al., 2013; Gosselin et al., 2016) producing spurious transcripts from intergenic regions (Struhl, 2007; Palazzo and Lee, 2015; Garland and Jensen, 2020; Palazzo and Koonin, 2020). Similarly, RNA transcripts are often mis-processed (Saudemont et al., 2017; Xu and Zhang, 2021). And even when they are not, mRNAs are frequently translated using the wrong start site and fail to terminate translation at the canonical stop codon (Kosinski and Masel, 2020; Xu and Zhang, 2020).

Of course, the ratio between canonical and non-canonical biological reactions could be increased by the expenditure of additional energy, thus increasing the difference between *E*_*1*_ and *E*_*2*_. For example, misincorporation of nucleotides is corrected by DNA repair enzymes and proof-reading machinery, both of which expend energy. However, if these non-canonical states are rare and only mildly deleterious, then there may not be enough benefit for the organism to invest in cellular machinery and energy which act to further drive the equilibrium toward the canonical state. Even if there is some benefit to pushing the equilibrium away from these deleterious states, there is diminishing returns as more and more energy is expended. However, probably the biggest obstacle, is the selective maintenance of genes that encode the additional machinery. Examples of this type of machinery are DNA repair enzymes and domains in polymerases responsible for proof-reading activity. The benefit of these genes, in terms of selection (*s*), must clear the drift barrier in order for natural selection to maintain them. It is for this very reason that the DNA mutation rate is strongly influenced by the effective population size (Lynch et al., 2016).

As long as the deleteriousness of these suboptimal activities remains below the critical threshold required for purging selection, they will not be eliminated and instead be subjected to evolution by drift (Lynch, 2007; Sung et al., 2012). With this in mind, it becomes obvious how cryptic transcription factor binding events or exotic RNA species can exist while serving no benefit (Palazzo and Gregory, 2014; Palazzo and Lee, 2015). Even tissue-specific expression will be subjected to unique noise given that each cell type expresses a unique set of transcription factors that activate distinct cryptic transcription start sites scattered throughout the genome (Levy et al., 2009; Palazzo and Lee, 2015). With the advent of powerful instruments that can identify rare and short-lived molecules, it should come as no surprise that we can document these non-adaptive entities. There is no doubt that further increasing the sensitivity of our detection instruments will reveal new biological insight, but it will also reveal an overwhelmingly large amount of cellular sloppiness.

## Global Solutions to Local Problems

If our cells are teeming with non-optimal reactions, mistake-riddled molecules, and non-adaptive processes, each being slightly deleterious, but not enough to be eliminated by natural selection, how do cells, and by extension multicellular organisms, manage to survive? This situation is exacerbated if the genome is constantly absorbing slightly deleterious mutations that increase messiness. One can consider each of these mutations as a local problem with an associated cost to the fitness of the organism. When we consider that natural selection operates on each individual mutation, the cost of a single mutation (or “local problem”) may not be sufficient for its elimination. Instead, it appears that eukaryotic cells have what is known as “global solutions” to these local problems (Rajon and Masel, 2011; Rajon and Masel, 2013; Koonin, 2016). The concept of global solutions shares many aspects with the idea of “buffering” systems advanced by Marc Kirschner and John Gerhart (Gerhart and Kirschner, 1997; Kirschner and Gerhart, 1998; Kirschner and Gerhart, 2005). It also shares many features with robustness advanced by other theorists (Félix and Wagner, 2008; Masel and Siegal, 2009). Global solutions are robust cellular mechanisms that maintain homeostasis. Often, these act to buffer not only environmental changes but also genetic changes. A good example would be chaperones, which not only prevent protein misfolding at high temperatures, but also promote the folding of proteins that have acquired destabilizing mutations. In the absence of sufficient chaperone activity, the effects of many such mildly deleterious mutations are revealed (Rutherford and Lindquist, 1998; Queitsch et al., 2002).

Other global solutions that increase the robustness of cells include RNA quality control mechanisms, such as non-sense mediated decay (Khajavi et al., 2006; Lykke-Andersen and Jensen, 2015), and the RNA export machinery, which retains mis-processed mRNAs and spurious transcripts in the nucleoplasm thus preventing their translation into potentially toxic proteins (Palazzo and Akef, 2012; Palazzo and Lee, 2015; Palazzo and Lee, 2018). Indeed, the evolution of the nucleus likely permitted the extensive mRNA processing that is characteristic of eukaryotic cells (López-García and Moreira, 2006; Martin and Koonin, 2006) and acts as a global solution to reduce the deleteriousness of countless RNA mis-processing events (Koonin, 2016) and the potential toxic effects of junk RNA (Palazzo and Lee, 2015; Palazzo and Lee, 2018). Besides non-sense mediated decay and mRNA export, the eukaryotic gene expression pathway is filled with distinct machineries that are coupled to each other allowing them to act as global solutions to errors and sub-optimal products (Maniatis and Reed, 2002; Warnecke and Hurst, 2011; Palazzo and Akef, 2012). For example, the splicing machinery directly deposits nuclear export factors on spliced mRNAs (Zhou et al., 2000; Luo et al., 2001; Masuda et al., 2005). For this reason, the splicing machinery is said to be coupled to the mRNA nuclear export machinery, and as a result spliced mRNA is more efficiently exported than mRNAs produced from intronless genes (Luo and Reed, 1999; Valencia et al., 2008). Since most protein-coding genes are well spliced, while spurious transcripts are not, the former but not the later are well exported (Palazzo and Lee, 2015). The gene expression pathway contains many other coupling reactions, and all of these promote the processing, nuclear export and translation of RNAs that contain features that are over-represented in protein-coding genes (“mRNA identity” features), while promoting the nuclear retention and degradation of spurious transcripts that lack these features (Palazzo and Akef, 2012; Palazzo and Lee, 2018; Palazzo and Kang, 2021). Overall, these systems act as global solutions and increase the robustness of the gene expression machinery.

Another important global solution is the piwi-associated RNA (piRNA) system which represses transposon activity in the germline of most eukaryotes (Czech et al., 2018). Instead of eliminating every transposon, the piRNA system can be mobilized to suppress the deleteriousness of most transposable elements that have an RNA intermediate in their life cycle. Indeed, it is believed that the piRNA, small interference RNA (siRNA), and micro RNA (miRNA) systems all evolved early in the evolution of eukaryotes to globally inhibit transposable elements and viruses (Shabalina and Koonin, 2008).

**FIGURE 2:**
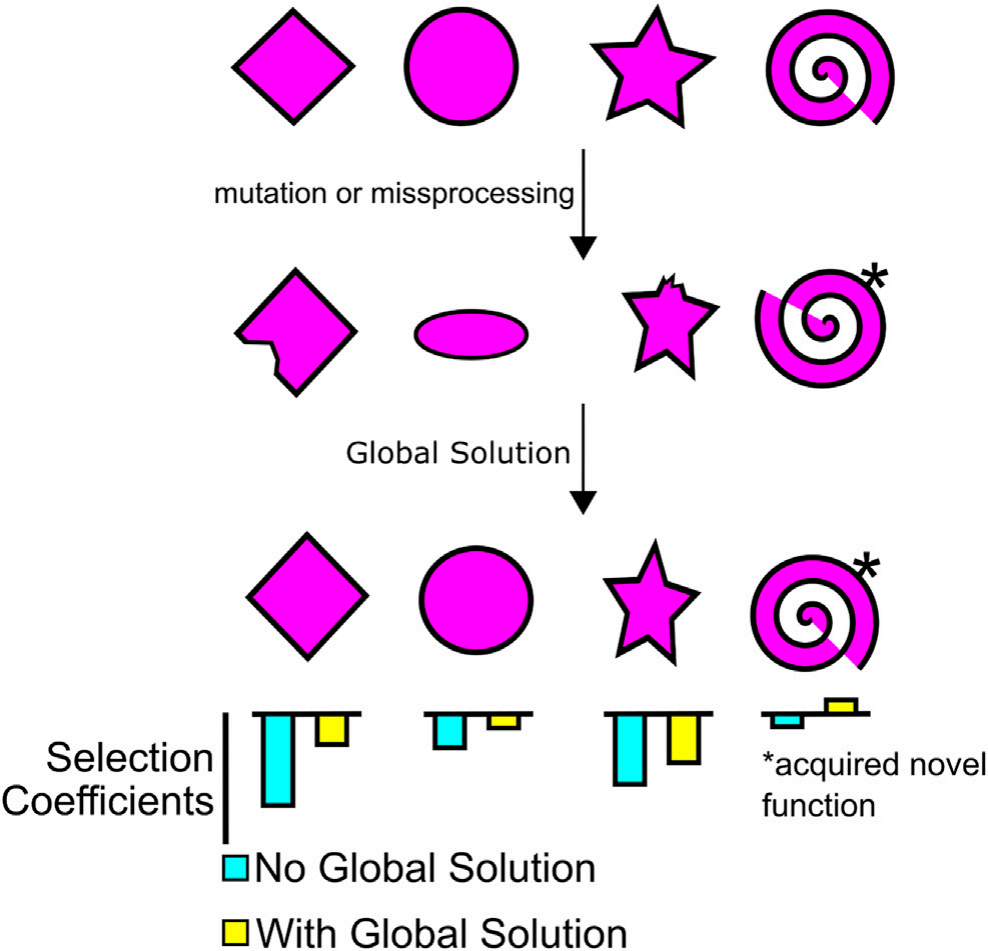
Global solutions reduce deleteriousness and promote evolvability.

The ultimate effect of these global solutions/buffering systems is to simultaneously elevate the selection coefficient of numerous mutations that share a common problem (Figure 2). Some of these mutations will have their selection coefficient values raised from slightly negative toward zero, and in some cases from near-zero to positive. For example, a novel mutation may allow an enzyme to perform an additional beneficial activity but also destabilize it. The proteostatic system may blunt the deleteriousness of any unfolded protein, while further promoting the beneficial activity. Non-sense mediated decay and coupling reactions in the gene expression system may eliminate misprocessed transcripts, while permitting some expression of an mRNA containing a poorly spliced novel exon. In this way, global solutions “encourage” the development of new innovations. Using similar arguments, Kirschner and Gerhart advocated that buffering systems facilitate phenotypic variation and promote “evolvability” (Gerhart and Kirschner, 1997; Kirschner and Gerhart, 1998; Kirschner and Gerhart, 2005). In a similar vein, many systems are likely present that increase the plasticity and phenotypic heterogeneity of organisms, and these appear to increase robustness (Rego et al., 2017; Mo et al., 2021).

## Mutational Bias

Although many evolutionary models assume that mutation supplies a random assortment of variants for natural selection to work on, mutation is not random, but is biased and this has major effects on genomic composition and the direction that evolution takes (Sueoka, 1962; Stoltzfus and Yampolsky, 2009; Nei, 2013). For example, in most organisms, transversion mutations (e.g., G to A mutations) are more common than transition mutations (e.g., G to C mutations). When amino acid differences between the proteomes of two closely related species are tabulated, the observed changes can be predicted by models that integrate mutation bias and the biochemical similarity of the swapped amino acids (Stoltzfus and Yampolsky, 2009). Ultimately, the nucleotide content of most genomes is in equilibrium, meaning that the nucleic acid composition is dictated by the interconversion rate between each of the bases (Sueoka, 1962).

Although the overall genomic content is dictated by global biases, mutational biases can also vary along the genome and likely dictate local nucleotide composition. For example, nucleosome occupancy can suppress cytosine deamination (Chen et al., 2012), and DNA curvature can alter local mutation rates (Duan et al., 2018). In addition, collisions between RNA polymerase and replication forks cause certain types of damage that are more prevalent at the start of genes (Aguilera, 2002; Lin and Pasero, 2012; Lin and Pasero, 2017). In transcription bubbles, the coding strand of transcribed genes is periodically single stranded and is thus more susceptible to certain types of DNA damage than the template strand. This likely leads to biased mutations in the coding strand of genes that are transcribed in the germ line, ultimately altering the frequency of all four nucleotides, and making these differ between the coding and template strand (Polak and Arndt, 2008; Hodgkinson and Eyre-Walker, 2011). These mutational biases may explain certain features of protein-coding genes, such as the elevated GC-content found at their 5’ ends (Palazzo and Kang, 2021).

Repair can also be biased. For example, transcribed genes undergo transcription coupled DNA repair which acts only on the template strand (Fousteri and Mullenders, 2008). DNA repair enzymes are more efficiently recruited to chromatin with certain histone modifications (Li et al., 2013; Supek and Lehner, 2017; Huang et al., 2018; Huang and Li, 2018). These biases likely explain why transcribed and intergenic DNA differ in their nucleotide content (Palazzo and Kang, 2021) and why highly expressed genes may have lower mutation rates than intergenic regions (Monroe et al., 2022). DNA repair polymerases also tend to have low fidelity, for example misincorporating G into A sites (McCulloch and Kunkel, 2008; Goodman and Woodgate, 2013). Thus, the repair of certain types of DNA damage may generate secondary mutations, at a lower rate, that have their own biases.

Bias also happens at the level of INDELs. In most organisms, small deletions are more common than small insertions (Kuo and Ochman, 2009; Sung et al., 2016). This bias may explain why prokaryotic genomes tend to have very little intergenic sequence (Mira et al., 2001; Moran et al., 2009). Tandem repeats also tend to be easily lost in prokaryotes due to recombination enzymes and this may further limit the growth of their genomes. In contrast, eukaryotes are parasitized by transposable elements which insert themselves into the genomes of their host, significantly increasing the rate of large insertions, and thus driving up genome size (Fedoroff, 2012; Palazzo and Gregory, 2014). Since the deleterious effects of these insertional mutations (i.e., local problems) are buffered by the eukaryotic gene expression machinery and the piRNA system (i.e., global solutions), this results in bloated genomes common to most eukaryotic lineages.

Other biases exist as well. DNA recombination during meiosis and some DNA repair pathways involve strand invasion between homologous chromosomes. Recombination between paternal and maternal chromosomes that differ due to the presence of heterozygous SNPs results in the formation of DNA with single nucleotide mismatches, which are then corrected in a biased manner to favor G:C base pairs over A:T base pairs. This process, called GC-biased gene conversion is quite prevalent in metazoans (Duret and Galtier, 2009). It has been estimated that each generation the average human genome gains roughly 100 *de novo* single nucleotide mutations (Chintalapati and Moorjani, 2020), and experiences 13 GC-biased gene conversion events (Paudel et al., 2020). When one considers that at most only 10% of all mutations occur in functional parts of the genome (Lindblad-Toh et al., 2011; Ward and Kellis, 2012; Rands et al., 2014; Graur, 2017), GC-biased gene conversion has at least as much influence over genomic content as selection, and this has been observed in the human genome (Pouyet et al., 2018). Ultimately, biased mutation (and biased repair) drastically shapes genomic features, directing the paths of both adaptive evolution and random genetic drift (Figure 1).

## The Importance of the Null Hypothesis

A key strength for the neutral theory of molecular evolution is that it provides molecular biologist with a null model for testing evolutionary hypotheses. Populations are perpetually evolving given the constant change in allele frequency and mutation rates. Therefore, it is important to understand whether these changes are due to stochastic versus adaptive processes. To invoke adaptive forces as the main driver of a molecular trait, one must demonstrate that the trait could not have arisen solely due to drift and biased mutations. In addition, organisms that have invested in global solutions that blunt the deleteriousness effects of messiness, will tolerate mutations that create a certain degree of messiness. This messiness will accumulate as long as it does not pose a burden above the drift barrier. To invoke adaptive forces as the main driver for the existence of some entity or some process, one must demonstrate that the trait is not simply a product of a messy organism (Figure 1).

To see how an understanding of modern evolutionary thinking can shed light into biology, we will briefly revisit the molecular clock and discuss our current understanding of this phenomenon. Although the rate of evolutionary change is constant for a given protein-coding gene in different lineages, it has been documented that different protein-coding genes have different clock rates. Over 2 decades ago, it was demonstrated that protein conservation in yeast species (i.e., how slow the clock runs for a given protein) correlates with its expression level (Pál et al., 2001). In contrast, conservation level had little correlation with other features, such as whether the protein was essential for viability, or any other measure of importance (Wang and Zhang, 2009).

How does one make sense of this observation? Adaptive models would presume that most changes are due to positive selection. The null model is that most change is due to neutral, or slightly deleterious mutations. If we assume that most mutations are nearly-neutral (the null hypothesis), why would these accumulate in lowly expressed genes? One possible explanation is that highly expressed genes cannot easily accommodate slightly deleterious mutations that marginally destabilize the folding of their encoded protein, as this would impose a greater burden on the protein homeostasis and folding machinery (Drummond et al., 2005). In contrast, the slight misfolding of lowly expressed proteins is more tolerable as it imposes less of a burden on protein homeostasis. Highly expressed proteins also contribute to a greater number of intermolecular interactions (in terms of the *types of interactors* and total *number of interactions*). As a result, small changes in these proteins would either disrupt a greater number of functional interactions, or promote a greater number of misinteractions (Levy et al., 2012; Yang et al., 2012; Mehlhoff et al., 2020). Global solutions can be used to blunt small changes, but are not as effective against big changes. As such, highly expressed genes are under greater evolutionary constraint and are more optimized in terms of their structural stability and/or interactions than lowly expressed genes, which can more easily rely on global solutions to fold properly. Although this “E-R anticorrelation” (Expression, Rate of evolution) is seen in all life forms (Zhang and Yang, 2015), and has somewhat been experimentally validated in yeast (Wu et al., 2022), it is likely that the reasons for this may differ in mammalian cells where constraints on highly expressed mRNAs, in terms of RNA folding, translation, and other aspects of RNA biology, may be more significant than in yeast or prokaryotes (Liao et al., 2006). Despite this, it is widely thought that the E-R anticorrelation is due to different levels of constraint and that any change is due to a greater tolerance for deleterious alterations in certain genes or their encoded proteins.

Concepts from molecular evolution, and the use of null models, have been used to understand the evolution of a wide array of entities and biochemical processes. This includes the evolution of 5′UTRs (Lynch et al., 2005), introns (Hong et al., 2006), RNA modifications (Liu and Zhang, 2018; Jiang and Zhang, 2019) and RNA processing (Melamud and Moult, 2009; Pickrell et al., 2010; Saudemont et al., 2017; Xu and Zhang, 2021). In all these studies, many of the observed phenomena have features that are consistent with the null hypothesis—that they are largely shaped by neutral evolution. When these produce undesirable products that are nevertheless present in organisms, their effects are small enough that they do not clear the drift barrier and are largely blunted by global solutions.

## Why Tolerance for Sloppiness Can Act as a Catalyst for the Evolution of Complexity

Although the nature of complexity is widely disputed, many observers have sought to define it in terms of a network or a “system”. The complexity of a system is correlated with the number of parts and the number of interactions between these parts (Hinegardner and Engelberg, 1983; McShea, 2000). It has been naively assumed by some that biological complexity is a direct product of natural selection (Ekstig, 2015). In other cases, it has been pointed out that when one starts off with a simple state (in this case a primitive ancestor) that evolves (i.e., has the capacity to change), then it is almost a certainty that some of its progeny will drift towards a more complex state, the so called “Drunkard’s Walk” model (Gould, 1996). Other commentators have noted that if an evolving organism is built of similar components, be it duplicated genes or similar groups of cells that form body parts, each individual component over evolutionary time will accumulate unique changes (either adaptive or neutral) that will eventually cause each part to drift away from its copies and cause the system as a whole to become more complex (McShea and Brandon, 2010). This effect, the so called “zero force evolutionary law”, does not require selection per se. Although the drifting apart of these components, may explain how a many-component system becomes complicated over time, it does not explain how new parts are generated. Furthermore, these drift models do not explain one of the main dichotomies that we see in life on earth: that by most definitions, eukaryotes are more complicated than prokaryotes.

What could explain the striking difference in the relative simplicity of prokaryotes and complexity of eukaryotes? One major difference appears to be how these two forms of life evolve. Put simply, prokaryotes experience high levels of selection pressure, while eukaryotes do not (Lynch, 2007). In organisms where selection pressure is extreme, superfluous activity is wasteful and effectively eliminated by natural selection (Lynch, 2006). In organisms with a low effective population size, these extra features are not purged but instead are allowed to persist for extended evolutionary time and their deleterious effects are instead buffered by global solutions. Even within eukaryotes, lower effective population sizes correlate with longer and/or more numerous intergenic regions, introns, cryptic transcriptional start sites and other non-functional genomic entities (Lynch, 2007). Within this surplus of non-functional activity lies the raw substrates for the evolution of new components. To rephrase this idea in terms used by other evolutionary theorists, a sloppy cellular environment full of junk contains many available substrates that can be tinkered with (Jacob, 1977; Jacob, 2001) and eventually exapted (Gould and Vrba, 1982) to form new functional parts. More recently, the detailed process of exaptation has been investigated, and surprisingly, the evolution of junk to functional entities often involves processes that do not rely on positive selection, but rather on neutral evolution.

## Constructive Neutral Evolution

As discussed above, non-functional entities are inefficiently eliminated in organisms that evolve under weak selection regimes. In the case of junk RNA, their persistence allows them to explore sequence space over considerable evolutionary time, allowing them to potentially acquire additional activities, or what is known as “excess capacity” (Stoltzfus, 1999). In some cases, the excess capacity overlaps the activity of some other functional entity. When situations like this arise, the activity of both new and existing parts, will mutationally decay to the point where either one activity is eliminated, or both activities become essential for organismal fitness. In this second scenario, there is an “accidental dependency” on the newly created entity (Stoltzfus, 1999). This is an example of the larger phenomenon, known as constructive neutral evolution (Stoltzfus, 1999; Stoltzfus, 2012). Note, that at no point was a new activity shaped by positive selection. Rather, the excess capacity was accidental and often a byproduct of proteins and RNA that have messy non-optimal activities. Furthermore, the eventual retention of the new part was due to mutational decay of the old part. At the end of this process the organism does not experience an increase in fitness. It however gains a new functional part and hence an increase in its complexity. An example of this is the evolution of junk RNA into functional long non-coding RNAs (Palazzo and Lee, 2018; Palazzo and Koonin, 2020). Eventually, these new functional entities may be further co-opted to generate entirely new evolutionary innovations and used to explore novel evolutionary trajectories. Organisms that experience strong selection regimes tend to eliminate messiness and as a result do not have as many raw substrates to fuel constructive neutral evolution. Organisms that experience weak selection regimes do not eliminate messiness. Instead, these new non-functional entities are allowed to explore sequence space for extended periods of time, increasing the chance that they accidentally evolve a new excess capacity.

As described above, an increase in complexity is not only due to the creation of new parts, but also through the establishment of new interactions between existing parts. Again, the rewiring of interaction networks is enhanced by constructive neutral evolution. For example, proteins that have an established function may acquire initial mutations that give it additional properties (i.e., excess capacity). As we pointed out earlier, enzymes and proteins are inherently messy and can promote many non-optimal reactions that may not be initially selected for. In some cases, these new activities can simply be the generation of a novel binding site between two unrelated proteins. Initially, these additional binding events may serve no function, but may allow the two proteins to structurally stabilize one another through non-specific chaperoning. These initial fortuitous mutations then allow for additional slightly deleterious mutations that structurally destabilize both proteins. The deleteriousness of these secondary mutations is of course blunted by the fact that the two proteins act as chaperones for each other. However, now the association of the two proteins, which originally was non-functional, is now required for their structural stability (i.e., accidental dependency). Like a molecular ratchet, the protein-binding interface becomes more and more essential as each complex member acquires more and more of these secondary destabilizing mutations. This type of constructive neutral evolution explains how cellular machineries, such as the ribosome and spliceosome, have gained components over evolutionary time (Stoltzfus, 1999; Gray et al., 2010; Lukeš et al., 2011; Stoltzfus, 2012; Linquist et al., 2020). This model has been experimentally validated in other multi-protein complexes and predicts the long-term evolution of amino-acid composition seen in many proteins that are part of large complexes (Hochberg et al., 2020).

## Summary

Most molecular biologists use an antiquated model of how evolution shapes biological processes leading them to an unrealistic hyper-adaptationalist view. A prime example of this is the interpretation of the ENCODE project results. Ultimately, this ultra-Darwinian mindset perpetuates the notion that the genome, and life itself, is like a Swiss watch—ornate, and complicated, with every part hand crafted for a specific purpose. This view is completely compatible with the idea that genome is pure information. However, this view is based on ignorance of developments in molecular evolution. It also ignores principles of biochemistry, that predict suboptimal reactions and widespread promiscuity. A more modern view of the eukaryotic cell, shaped by drift-dominated evolution, is a messy junk-filled entity, full of Rube-Goldberg contraptions that were hobbled together by non-adaptive forces. With this new vantage point, certain aspects of eukaryotic biology become clarified, including the evolution of complexity.
